# Supplementary material for: Advanced sulfide solid electrolyte by core-shell structural design
Source: Nat Commun. 2018 Oct 2;9:4037. doi: 10.1038/s41467-018-06123-2 (PMC6168527; doi:10.1038/s41467-018-06123-2)
Supplement: Supplementary file 1 — Supplementary information [file 41467_2018_6123_MOESM1_ESM.pdf]

Supplementary Information

Wu et al.

Advanced Sulfide Solid Electrolyte by Core–Shell Structural Design

Supplementary Figures

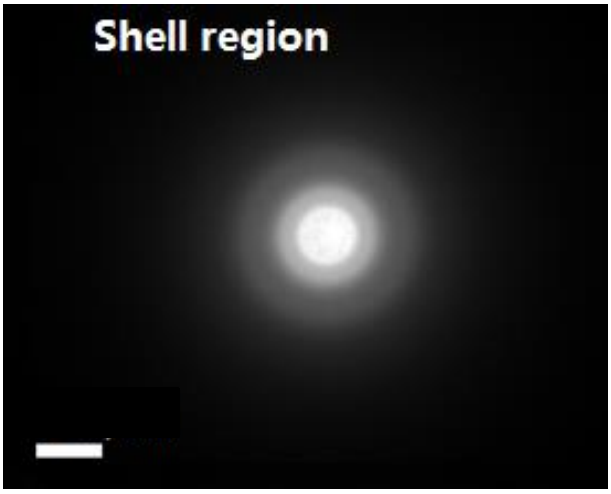

Supplementary Figure 1| TEM diffraction pattern on the shell of LSPS solid electrolyte (LSPS460). Results show the amorphous structure. Scale bar in the figure stands for 5 1/nm.

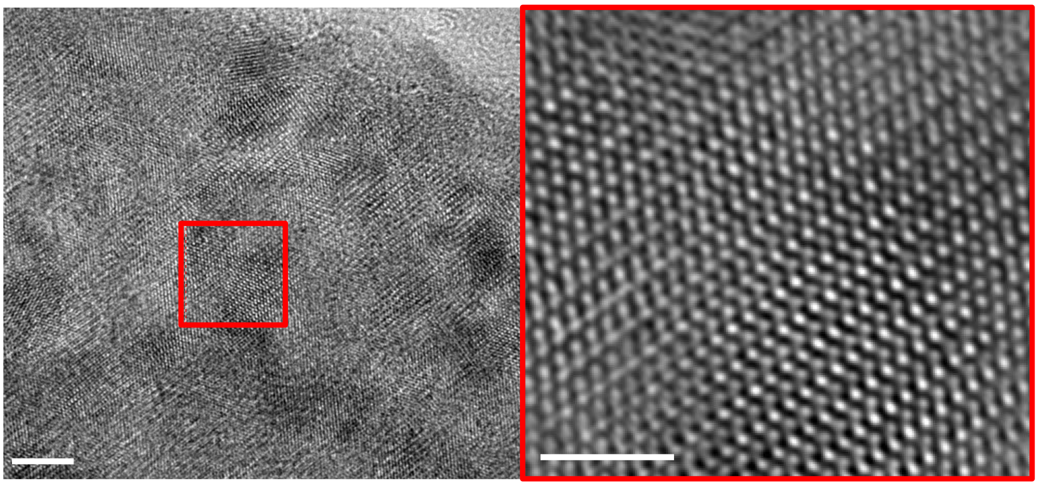

Supplementary Figure 2| Typical HRTEM images of particles inside the amorphous shell of LSPS 450. Results show that they are single－crystals embedded in the amorphous shell. Scale bars in the left and right figure stand for 5 and 2 nm, respectively.

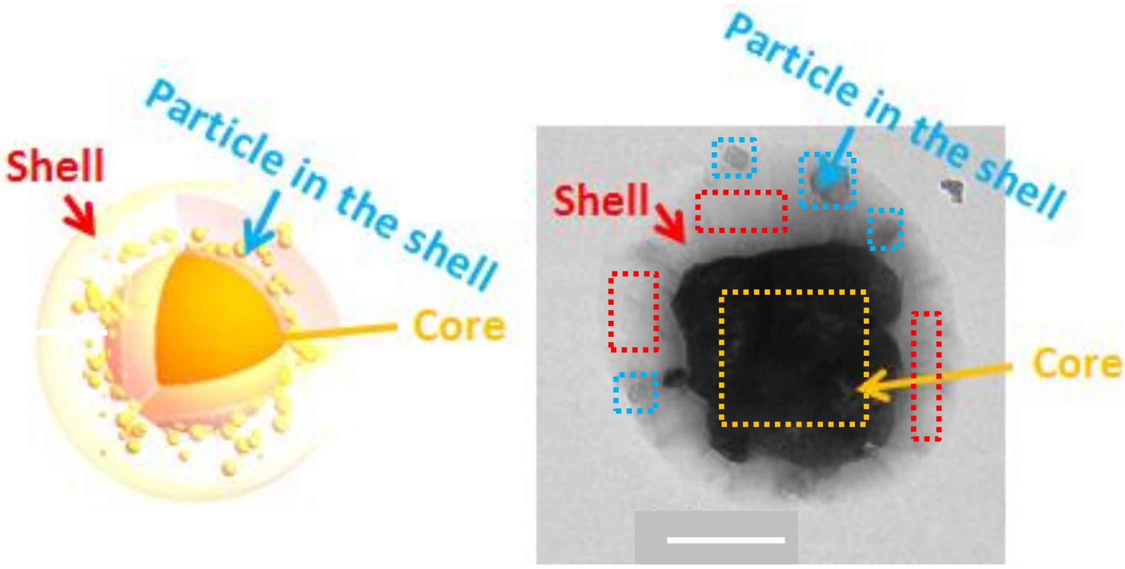

Supplementary Figure 3| Illustration of the core–shell structure and EDS signal source of LSPS solid electrolyte particles. Multiple areas inside different particles were chosen for STEM EDS signal acquisition and analyses for each sample. For the particle shown in this TEM bright-field Figure (as an example), 3 different areas of the shell, 4 different particles inside the shell and the whole core area were chosen for EDS signal acquisition. Similar procedure was performed on multiple particles for each LSPS sample, and the average values of the obtained data were statistically analyzed and summarized in Figure 2 c and d in the main text. Scale bars in the right figure stand for 0.8 μm.

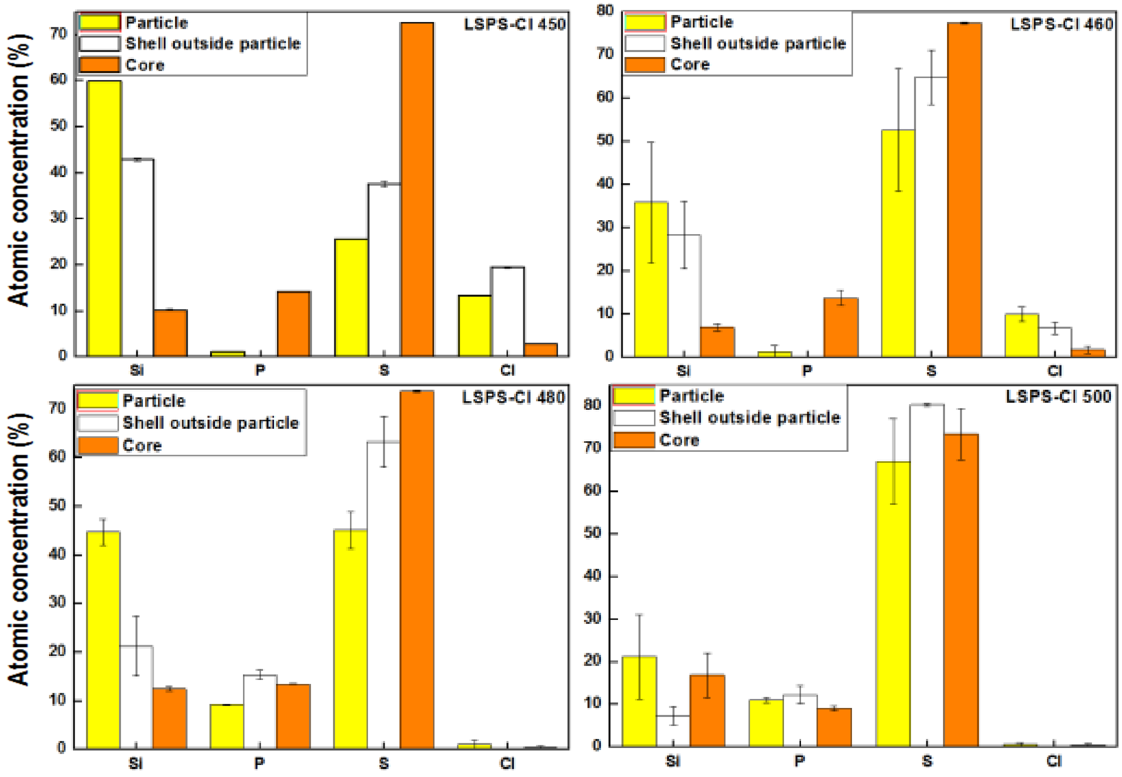

Supplementary Figure 4| STEM EDS analyses of LSPS 400, 450, 480 and 500. Results show that all regions of these particles are comprised of Si, P, S and Cl. Lithium cannot be detected by EDS technique, due to its small atomic weight and number.

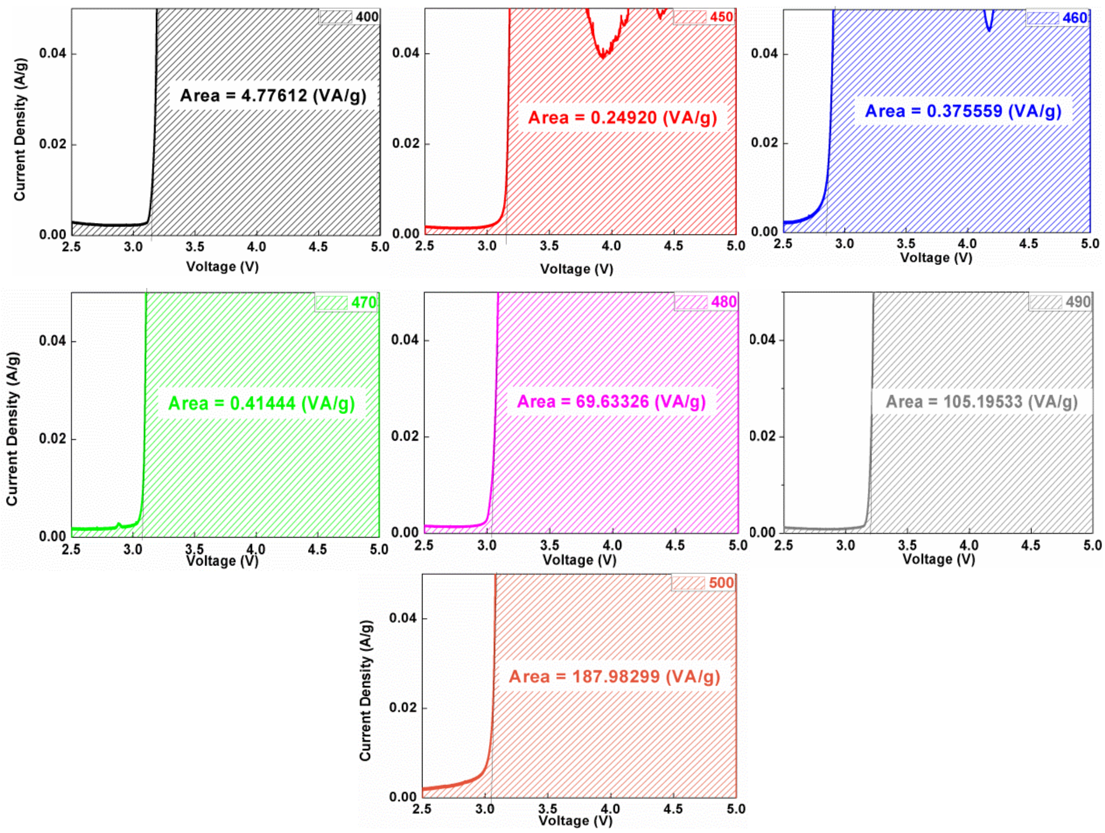

Supplementary Figure 5| The onset voltages of decomposition for all 7 LSPS samples annealed at different temperatures. They were obtained by drawing tangent lines for the horizontal base and the current density curve for each sample.

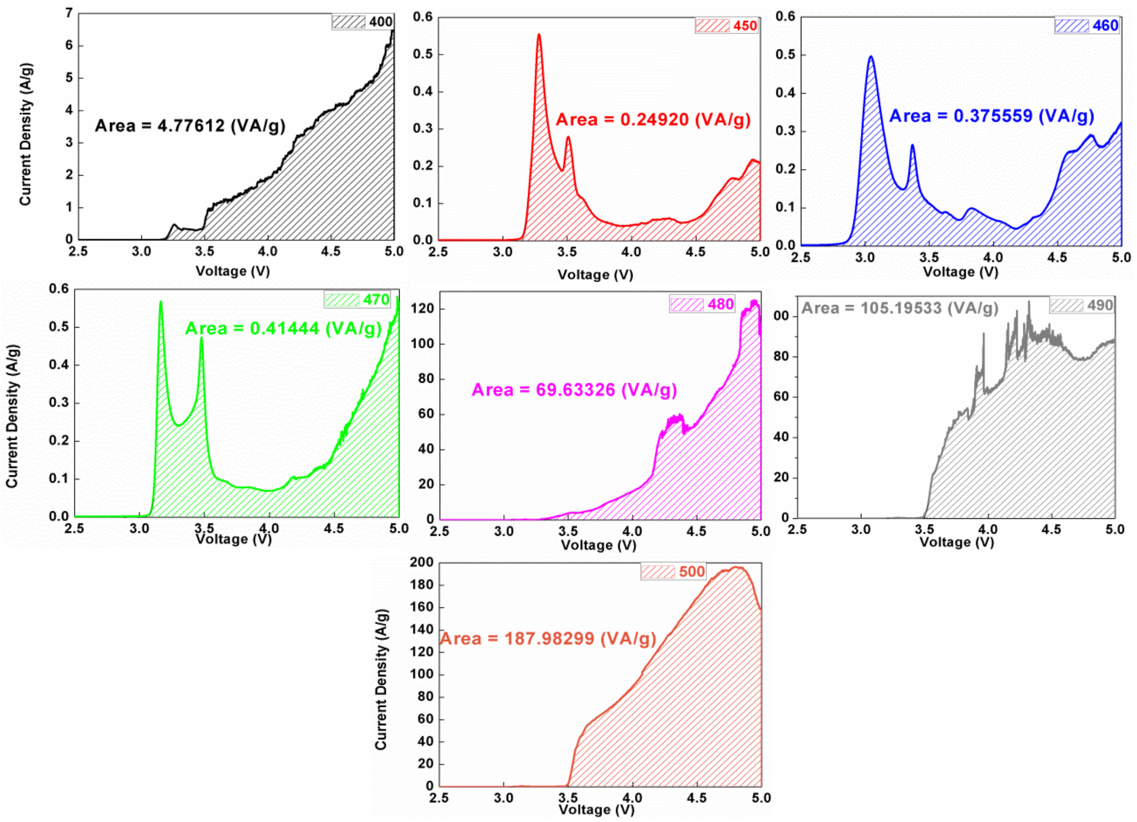

Supplementary Figure 6| The integral spectrum intensity of decomposition for all 7 LSPS samples annealed at different temperatures. The integral spectrum intensity of decomposition was obtained by integrating the shadowed area between the current density curve and the x axis for each sample.

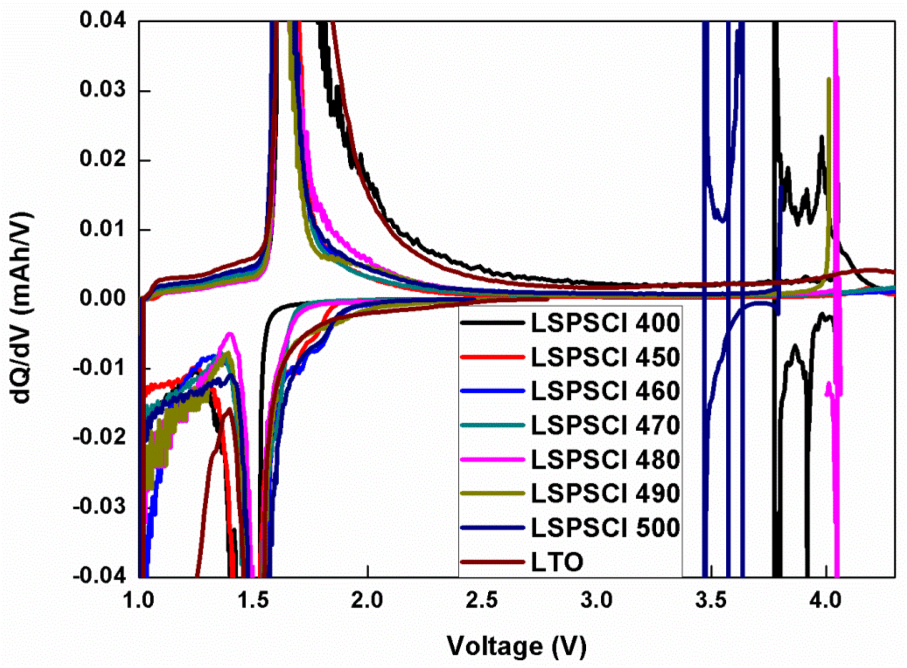

Supplementary Figure 7| Derivative of capacity versus voltage (dQ/dV) plots for $Li_4Ti_5O_{12}$+LSPS+C/LSPS/glass fiber/Li cells incorporating different LSPS materials and $Li_4Ti_5O_{12}$/LSPS/glass fiber/Li cells. Results show that the peaks at high voltages are characteristic peaks caused by LSPS materials, not relevant to $Li_4Ti_5O_{12}$.

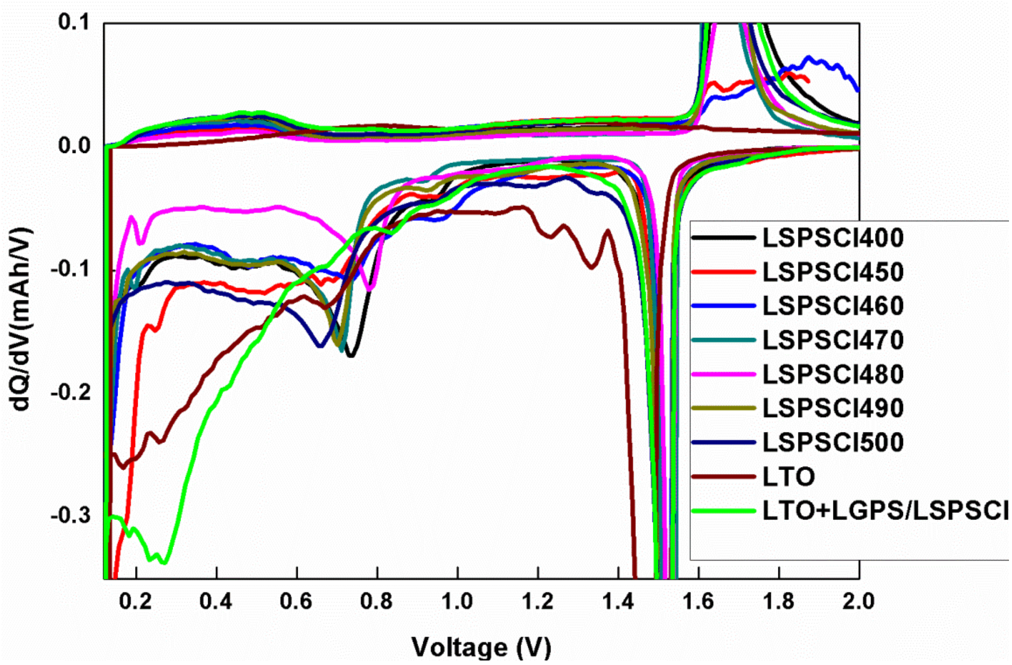

Supplementary Figure 8| Derivative of capacity versus voltage (dQ/dV) plots for $Li_4Ti_5O_{12}$+LSPS+C/LSPS/glass fiber/Li cells incorporating different LSPS materials and $Li_4Ti_5O_{12}$+LGPS/LSPS470/glass fiber/Li cells. Results show that the $Li_4Ti_5O_{12}$+LGPS/LSPS470/glass fiber/Li cell has no peak at 0.7V. In other words, the 0.7V peak is not caused by the liquid electrolyte or the interaction between LSPS and liquid electrolyte.

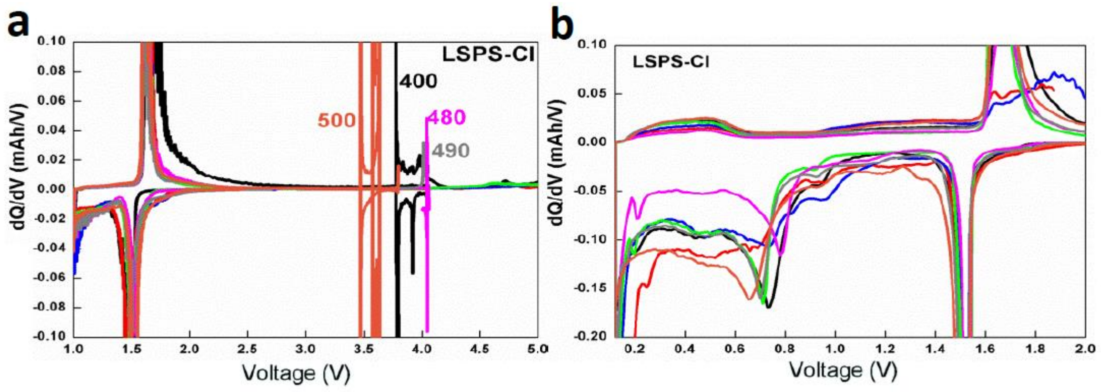

Supplementary Figure 9| Derivative of capacity versus voltage plots for 7 batteries cycled between high and low voltage ranges. (a) 1–5V; (b) 0.1–2V.

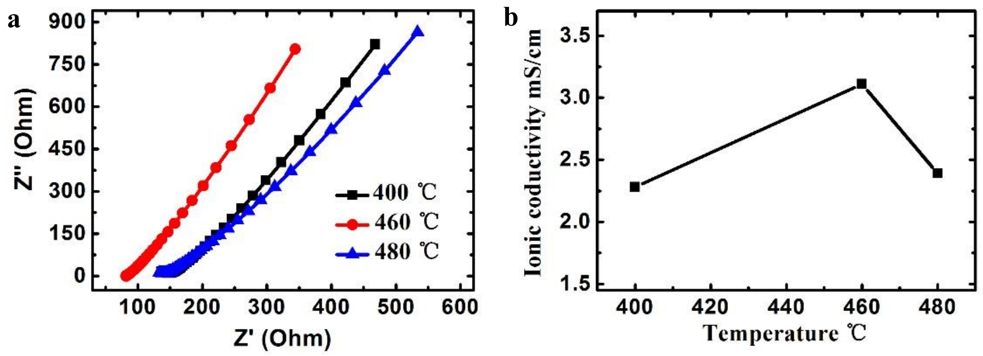

Supplementary Figure 10| Impedance profiles and ionic conductivities of LSPS solid electrolytes annealed at different temperatures. (a) Impedance profiles of C–LSPS/LSPS/C–LSPS with electrolytes at different annealing temperatures tested under a low pressure of 1 MPa. The total thicknesses of the cells are 800–900 μm and the C–LSPS layer is thin (~2% of total thickness) so that the profiles reflects the impedance behavior close to the real solid state electrolyte. (b) Ionic conductivities of LSPS with different annealing temperatures derived from panel a.

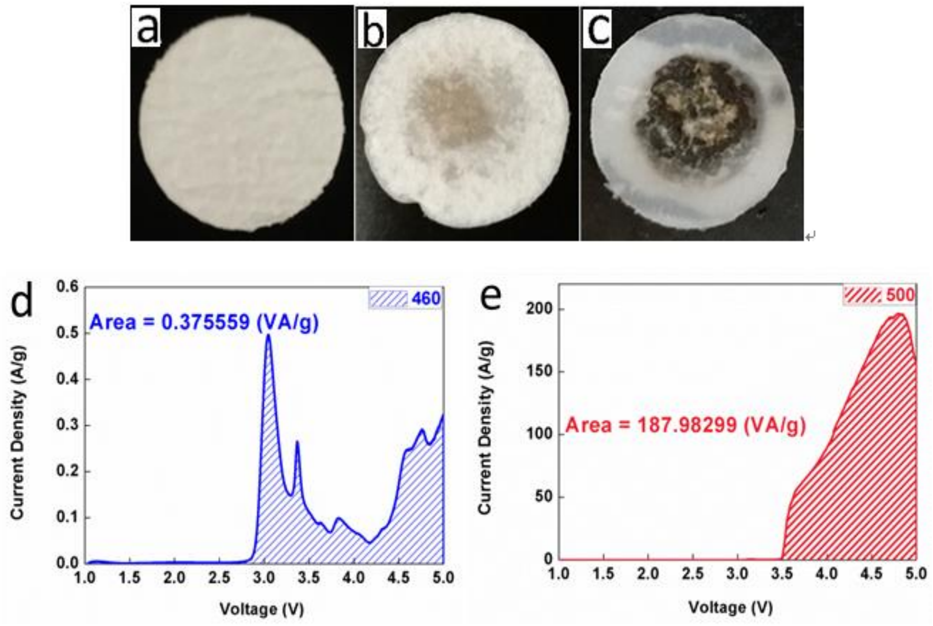

Supplementary Figure 11| CV test results of different LSPS solid electrolytes and separator photos after these CV tests. (a) Original separator; (b) Separator in the battery for minor–decomposition material (LSPS460) after CV test between 1V and 5V. (c) Separator in the battery for severe–decomposition material (LSPS500) after CV test between 1V and 5V. (d) CV test result for minor–decomposition material (LSPS460) between 1V and 5V. (e) CV test result for severe–decomposition material (LSPS500) between 1V and 5V.

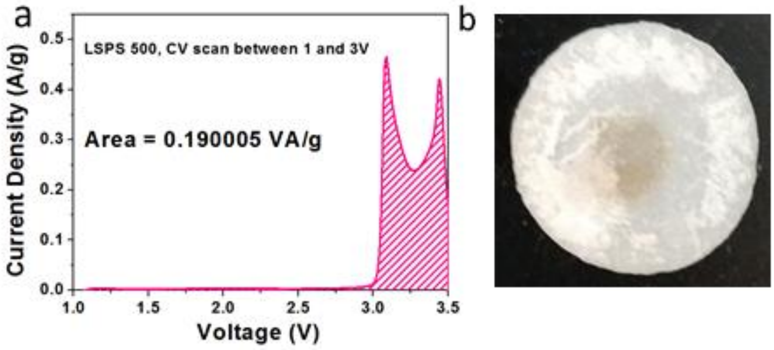

Supplementary Figure 12| CV test result of LSPS500 and separator photo after CV test. (a) CV test result for severe–decomposition material (LSPS500) between 1V and 3.5V to avoid the high voltage decomposition. (b) Picture of separator in the battery for severe–decomposition material (LSPS500) after CV test between 1V and 3.5V.

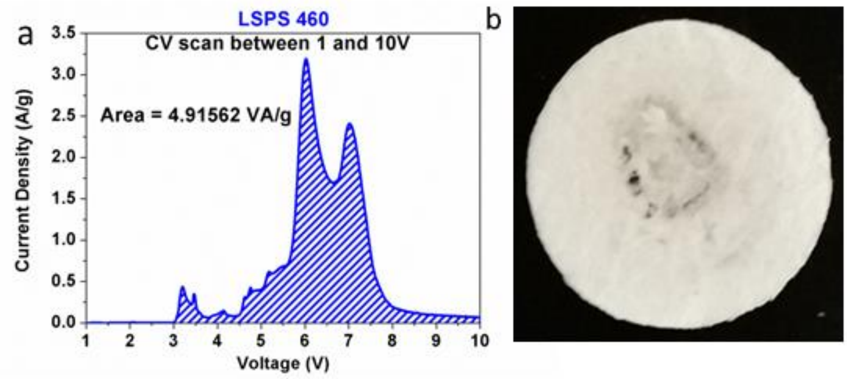

Supplementary Figure 13| CV test result of LSPS460 and separator photo after CV test. (a) CV test result for minor–decomposition material (LSPS460) between 1V and 10V. (b) Picture of separator in the battery for minor–decomposition material (LSPS460) after CV test between 1V and 10V.

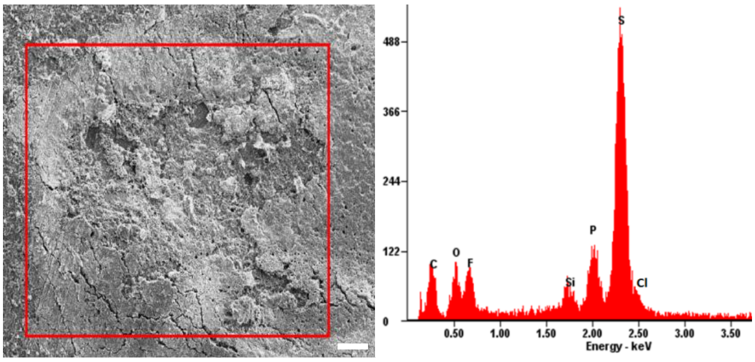

Supplementary Figure 14| SEM image and energy dispersive spectrum of the black region on the separator in Supplementary Figure 11c. Scale bar in the left figure stands for 200 μm.

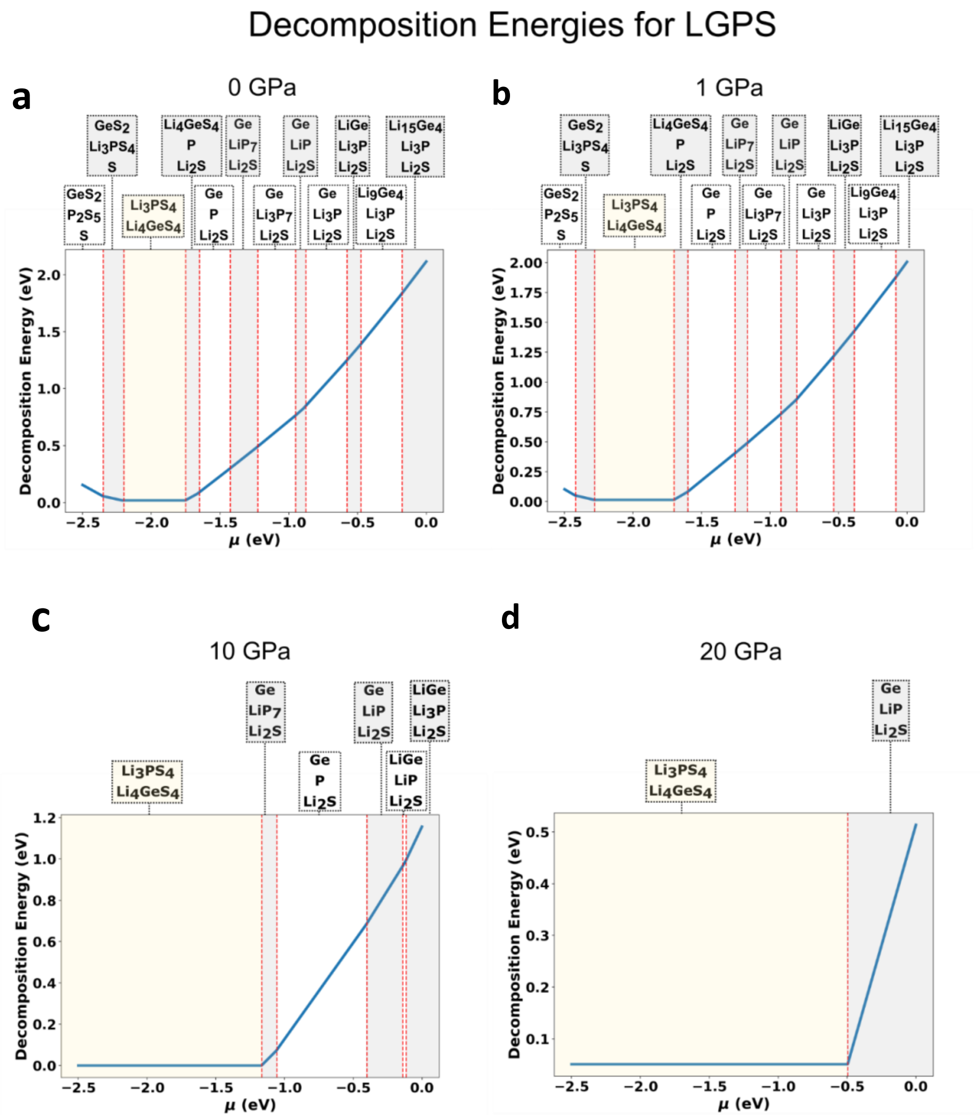

Supplementary Figure 15| Decay energies and electrochemical stability windows for LGPS at varying levels of applied pressure. The set of compounds above each partition indicate which decay pathway is dominant for LGPS in that electrochemical potential region and the y axis plots with how much energy that decay will proceed. The yellow shaded region indicates at which chemical potentials LGPS is stable at room temperature. **(a)** At no applied pressure, the voltage window is 1.75 to 2.2 V **(b)** 1 GPa of applied pressure increases the voltage window slightly to 1.7 to 2.28 V. **(c)** By 10 GPa the upper threshold has exceeded 2.5V and the lower is at 1.75 V **(d)** Finally, at 20 GPa of applied pressure, the LGPS is stable from 0.495 V to in excess of 2.5V. (LGPS is chosen here for calculation because the free energy Φ for all possible decay products of LGPS can be obtained from various material databases).

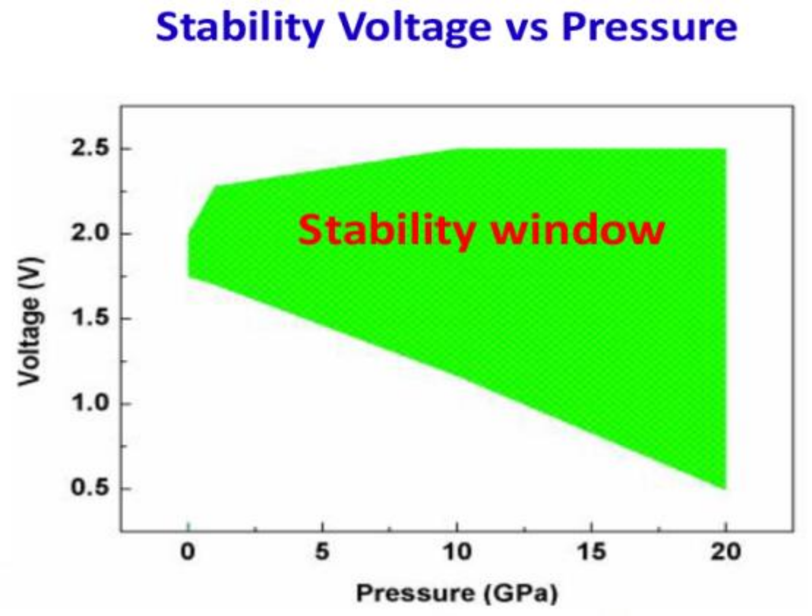

Supplementary Figure 16| The stability voltage window of LGPS as a function of various pressures from 0–20GPa. LGPS is chosen here for calculation because the free energy Φ for all possible decay products of LGPS can be obtained from various material databases.

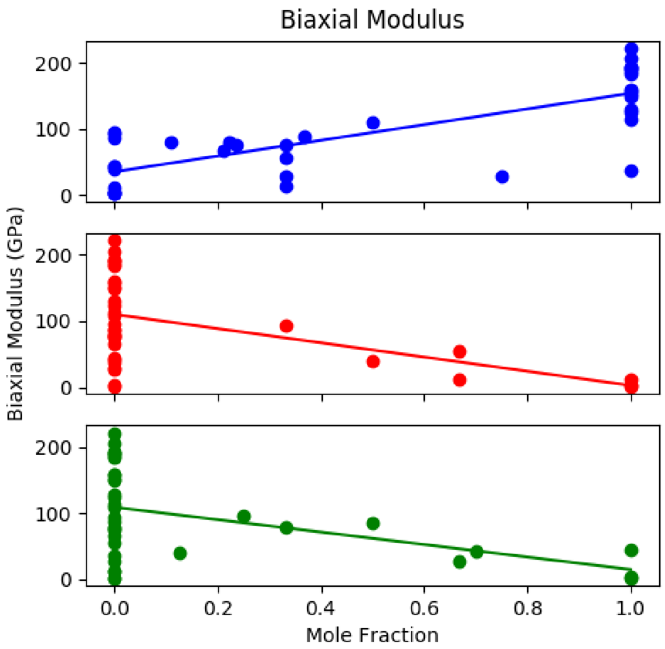

Supplementary Figure 17| Biaxial modulus (GPa) and Poisson ratio and trendlines v.s. mole fraction of different elements. Si (top), S (middle), and P (bottom). Data points come from the Materials Project.

Supplementary Tables

Supplementary Table 1| Summary of the decomposition voltages and severities of LSPS–Cl samples annealed at various temperatures derived from CV curves

Sample	Low–voltage Peak (V)	High–voltage Peak: Onset decomposition voltage (V)	High–voltage Peak: Integrated Current (VA/g)	Decomposition Degree
**400**	0.66	3.15	4.77612	**Medium**
**450**	0.66	3.16	0.24920	
**460**	0.68	2.87	0.37556	**Minor**
**470**	0.71	3.09	0.41444	
**480**	0.69	3.04	69.63326	
**490**	0.67	3.20	105.19533	**Severe**
**500**	0.68	3.06	187.98299	

Supplementary Table 2| H(p) for each pressure and material plotted in Supplementary Figure 11.

System	H(0GPa)/eV	H(1GPa)/eV	H(10GPa)/eV	H(20GPa)/eV
Ge	−4.487796E+00	−4.338350E+00	−3.079492E+00	−1.803821E+00
GeS	−9.148459E+00	−8.882685E+00	−6.765160E+00	−4.691484E+00
GeS_2	−1.369779E+01	−1.317950E+01	−9.331516E+00	−6.359788E+00
Li_2S	−1.196982E+01	−1.168203E+01	−9.323823E+00	−7.015825E+00
Li_3P	−1.391181E+01	−1.354894E+01	−1.057828E+01	−7.679334E+00
Li_3PS_4	−3.509377E+01	−3.406774E+01	−2.613501E+01	−1.958275E+01
Li_4GeS_4	−3.818960E+01	−3.713570E+01	−2.861505E+01	−2.036525E+01
$Li_4P_2S_6$	−5.372166E+01	−5.240342E+01	−4.165673E+01	−3.110949E+01
$Li_{15}Ge_4$	−5.202579E+01	−5.012603E+01	−3.529954E+01	−2.133391E+01
LGPS	**−1.080921E+02**	**−1.050711E+02**	**−8.132142E+01**	**−5.876544E+01**
P	−5.378355E+00	−5.252024E+00	−4.295360E+00	−3.397391E+00
S	−4.061113E+00	−3.889834E+00	−2.767311E+00	−1.745577E+00
P_2S_5	−3.260306E+01	−3.135725E+01	−2.295823E+01	−1.531866E+01
LiP_7	−4.097063E+01	−3.990250E+01	−3.163096E+01	−2.382346E+01
Li_3P_7	−4.721150E+01	−4.599375E+01	−3.635586E+01	−2.774939E+01
LiP	−8.365624E+00	−8.172500E+00	−6.580108E+00	−5.014365E+00
LiGe	−6.956550E+00	−6.756500E+00	−5.100235E+00	−3.458691E+00
Li_9Ge_4	−3.962443E+01	−3.836025E+01	−2.812853E+01	−1.824771E+01

Supplementary Table 3| Quantitative SEM EDS analysis of the spectrum in Supplementary Figure 15 for all the detected elements from the black region on the separator of Supplementary Figure 12.

Element	Wt%	At%
**CK**	49.98	65.63
**OK**	13.27	13.08
**FK**	08.94	07.42
**SiK**	01.82	01.02
**PK**	04.62	02.35
**SK**	21.00	10.33
**ClK**	00.36	00.16

Supplementary Notes

The following experiments were performed to show the oxidation/decomposition products of LSPS solid electrolytes, and more importantly, to show that the minor–decomposition LSPS have true larger electrochemical stability:

a. We disassembled the batteries after CV tests between 1V and 5V and took the photos of the separators. The color of separator remains almost unchanged (Supplementary Figure 11b) for minor–decomposition material (such as LSPS460), while for severe–decomposition material (such as LSPS500) the separator becomes black (Supplementary Figure 11c). Compositional analyses on the black region will be discussed later.

b. We performed the CV scan of another battery in a narrower voltage range between 1 and 3.5 V to avoid the decomposition current peak between 3.5V and 5V for severe–decomposition material (LSPS500) This time, the separator remains to be white (Supplementary Figure 12b)

c. To explore the true electrochemical stability of our stable/minor–decomposition material (LSPS460) at high voltages, we expanded the CV test window from 1–5V to 1–10V, where the integrated current density is 4.91562 VA/g (Supplementary Figure 13a). The value is still much smaller than that of severe–decomposition material (LSPS500) scanned within a much narrower window of 1 and 5V (187.98299 VA/g). The corresponding separator after the CV scan between 1 and 10V (Supplementary Figure 13b) remains almost white with some very small black regions, indicating a small–degree of decomposition of LSPS.

d. To further confirm that the black color on separator in Supplementary Figure 11c is due to decomposition of LSPS, we performed SEM EDS analyses on the black region of the separator (Supplementary Figure 14), which shows the existence of Si, P, S and Cl with their composition summarized in Supplementary Table 3. We can easily conclude that at least S should be from the decomposition of LSPS.

Extended Derivation

Main text Equation 2 defines the effective compressibility of the shell, which can be used to express the volume constriction of the core material at low pressures as equation 1.

$$V_{core}(p) \approx V_{core}^0 + \beta_{shell} V_{core}^0 p \qquad (1)$$

Where $V_{core}^0 = V_{SE}(p=0)$ is the volume of the solid electrolyte core prior to any decomposition/pressurization. Combining with main text Equation 5:

$$x_d V_d(p) + x_{SE} V_{SE}(p) = V_{core}(p) = V_{core}^0 + \beta_{shell} V_{core}^0 p \qquad (2)$$

Taylor expanding V_d, V_{SE} about zero pressure gives $V_i(p) \approx V_i^0 - \beta_i V_i^0 p$, which can be substituted into Equation 2.

$$x_d V_d^0 + x_{SE} V_{SE}^0 - p(x_d \beta_d V_d^0 + x_{SE} \beta_{SE} V_{SE}^0) = V_{SE}^0 + \beta_{shell} V_{SE}^0 p \qquad (3)$$

Using the fractional relation $x_d + x_{SE} = 1$.

$$x_d V_d^0 + (1 - x_d) V_{SE}^0 - p(x_d \beta_d V_d^0 + (1 - x_d) \beta_{SE} V_{SE}^0) = V_{SE}^0 + \beta_{shell} V_{SE}^0 p \qquad (6)$$

$$x_d (V_d^0 - V_{SE}^0) - p(x_d \beta_d V_d^0 + (1 - x_d) \beta_{SE} V_{SE}^0) = \beta_{shell} V_{SE}^0 p \qquad (7)$$

$$x_d (V_d^0 - V_{SE}^0) = \left(\beta_{shell} V_{SE}^0 + (x_d \beta_d V_d^0 + (1 - x_d) \beta_{SE} V_{SE}^0)\right) p \qquad (8)$$

$$x_d (V_d^0 - V_{SE}^0) = \left(\beta_{shell} V_{SE}^0 + \beta_{SE} V_{SE}^0 + x_d (\beta_d V_d^0 - \beta_{SE} V_{SE}^0)\right) p \qquad (9)$$

$$\rightarrow p = \frac{x_d (V_d^0 - V_{SE}^0)}{V_{SE}^0 (\beta_{shell} + \beta_{SE}) + x_d (\beta_d V_d^0 - \beta_{SE} V_{SE}^0)} \qquad (10)$$

Restricting focus to the onset of decay when $x_d \ll 1$, Equation 10 becomes:

$$p \approx \frac{x_d (V_d^0 - V_{SE}^0)}{V_{SE}^0 (\beta_{shell} + \beta_{SE})} = \frac{x_d \epsilon_{RXN}}{(\beta_{shell} + \beta_{SE})} \rightarrow \quad x_d(p) = p \frac{(\beta_{shell} + \beta_{SE})}{\epsilon_{RXN}} \qquad (11)$$

The decay energy at given chemical potential defined by main text equation 4 becomes:

$$\Delta\phi_V(\mu) = x_{SE}\, \phi_{SE}(p, \mu) + x_d\, \phi_d(p, \mu) - \phi_{SE}(0, \mu) \qquad (12)$$

$$\Delta\phi_V(\mu) = (1 - x_d) \phi_{SE}(p, \mu) + x_d\, \phi_d(p, \mu) - \phi_{SE}(0, \mu) \qquad (13)$$

$$\Delta\phi_V(\mu) = x_d \left(\phi_d(p, \mu) - \phi_{SE}(p, \mu)\right) + \phi_{SE}(p, \mu) - \phi_{SE}(0, \mu) \qquad (14)$$

In order for the decay given above to proceed, Equation 14 must be negative. Recognizing the first term as $\Delta\phi(p, \mu) = \phi_d(p, \mu) - \phi_{SE}(p, \mu)$ as the isobaric decay:

$$x_d\, (\phi_d(p, \mu) - \phi_{SE}(p, \mu)) + \phi_{SE}(p, \mu) - \phi_{SE}(0, \mu) < 0 \qquad (15)$$

$$\Delta\phi(p,\mu) < -\frac{\phi_{SE}(p,\mu) - \phi_{SE}(0,\mu)}{x_d} \tag{16}$$

Again restricting focus to the onset of decay where $p \to \delta p$ and x_d is given by Equation 11, Equation 16 becomes:

$$\Delta\phi(\delta p,\mu) < -\frac{\epsilon_{RXN}}{\beta_{shell} + \beta_{SE}}\frac{\delta\phi_{SE}}{\delta p} = -\frac{\epsilon_{RXN}}{\beta_{shell} + \beta_{SE}}V_{core} \tag{17}$$

Defining an effective bulk modulus for the core–shell microstructure $K_{eff} \equiv (\beta_{shell} + \beta_{SE})^{-1}$, the restriction on the isobaric decay energy density such that micro–structure decay can occur is given by Equation 18.

$$\frac{|\Delta\phi(\delta p,\mu)|}{V_{core}} \approx \frac{|\Delta\phi(0,\mu)|}{V_{core}} > K_{eff}\epsilon_{RXN} \tag{18}$$
